# Percutaneous Balloon Mitral Valvuloplasty in Children and Adolescents With Juvenile Rheumatic Mitral Stenosis: Single Centre Experience

**DOI:** 10.7759/cureus.84707

**Published:** 2025-05-23

**Authors:** Abhay Pota, Tarun Parmar, Priyanka Agarwal, Bhavik Champaneri, Amit Mishra, Trushar Gajjar, Jigar Surti, Shilpa Deodhar, Amit Kungwani, Gajendra Dubey

**Affiliations:** 1 Department of Pediatric Cardiology, UN Mehta Institute of Cardiology and Research Centre (UNMICRC), Ahmedabad, IND; 2 Department of Cardiovascular Thoracic Surgery, UN Mehta Institute of Cardiology and Research Centre (UNMICRC), Ahmedabad, IND; 3 Department of Anesthesia, UN Mehta Institute of Cardiology and Research Centre (UNMICRC), Ahmedabad, IND; 4 Department of Cardiology, UN Mehta Institute of Cardiology and Research Centre (UNMICRC), Ahmedabad, IND

**Keywords:** balloon sizing strategy, juvenile mitral stenosis, mitral regurgitation, percutaneous balloon mitral valvuloplasty (pbmv), retrospective study

## Abstract

Background

Rheumatic mitral stenosis (MS) in India is known to affect at a younger age, worsen rapidly, and have severe valvar and subvalvar pathology. Surgical interventions involve the risk of needing to redo surgery in the future, in addition to the acute risks of surgery itself and cardiopulmonary bypass. Therefore, percutaneous balloon mitral valvuloplasty (PBMV) has become an attractive alternative option, especially in children who rarely have any significant calcifications.

Method

We report our experience of PBMV in this retrospective study of 46 patients, aged 9 to 17 years (23 males, 23 females), with all being in the New York Heart Association (NYHA) class II/III. Seven patients had significant subvalvar pathology. All patients had severe pulmonary arterial hypertension (PAH). We used Accura balloons (Accura Medizintechnik GmbH, Karben, Germany) and estimated balloon size based on the height formula and used balloons 1-3 mm smaller than those derived from the formula.

Results

We used a smaller balloon in all the patients and had to upgrade in only seven cases. There was a significant reduction in the transmitral gradient from 19.93±6.21 mmHg to 3.54±1.1 mmHg (p<0.001) and a significant increase in the area of the mitral valve from 0.75±0.18 cm^2^ to 1.6±0.2cm^2^ (p<0.001). There was immediate symptomatic improvement in all patients, and there was no mortality. Moderate mitral regurgitation (MR) developed in six patients, one patient developed severe MR, and only one of them had subvalvar pathology.

Conclusion

Our results indicate that PBMV is an effective procedure in the younger population with juvenile MS. PBMV performed with undersized balloons (1-3 mm smaller than the height-based estimate) demonstrated comparable efficacy and safety, even in patients with subvalvular pathology.

## Introduction

Rheumatic heart disease continues to be a significant health burden in low and middle-income countries. Rheumatic mitral stenosis in the younger age group <20 years, also known as juvenile mitral stenosis, a term coined by Dr SB Roy from India, presents with unique disease characteristics [1]. While on one hand, these patients often have a rapidly progressive course, tend to have severe valvar and sub valvular deformity, present in advanced functional class and have evidence of severe pulmonary hypertension; while on the other hand, they continue to maintain sinus rhythm, are unlikely to have thrombus in left atrium, there is less incidence of valve calcification and are less likely to have thromboembolic events.

Surgical and percutaneous management strategies have both been reported to successfully treat this condition, but while surgical mitral valve replacement entails a lifelong risk of prosthetic heart valve thrombosis and also of reintervention after 10-15 years, percutaneous balloon mitral valvuloplasty (PBMV) has significantly lesser risks and also been proven to be as effective in children, as in adults, if not more [2]. We hypothesized that using a balloon undersized by 1-3 mm relative to the height-based formula would maintain efficacy while reducing the incidence of significant mitral regurgitation (MR).

The objective of this study was to evaluate the efficacy and safety of PBMV in juvenile MS and to determine whether a reduction in the size of the balloon used can reduce the incidence of MR.

## Materials and methods

This was a single-center retrospective cross-sectional study that included consecutive patients with severe mitral valve stenosis aged <18 years who had undergone balloon mitral valvotomy between January 2017 and June 2024 at our institute. Ethical approval for the study was given by an institutional ethics committee (UNMICRC/CARDIO/2023/27). The case files of all eligible patients were retrieved from the medical records department of our institute. All relevant variables, such as demographic details, clinical and diagnostic data, and cardiac catheterization procedure details, were recorded.

We measured mitral valve area (MVA), left atrial (LA) dimensions, trans mitral peak and mean pressure gradient, systolic pulmonary artery pressure (SPAP), and presence of MR and its mechanisms. All measurements were done pre- and post-procedure by two experienced pediatric cardiologists as per ASE guidelines. Differing measurements were resolved by internal blinded review by a third experienced pediatric cardiologist.

Severity of MR was determined by expressing the ratio of the maximal jet area to the left atrial area in the same view using colour flow mapping and graded from one to four according to Essop et al [3]. The procedure was deemed successful when the MV area was increased by >50%, and the final MV area was >1 cm2/m2 with MR not more than moderate.

Exclusion criteria

Our exclusion criteria for the PBMV were (a) a thrombus in the left atrium or left atrial appendage, (b) pre-existing mitral regurgitation severity more than mild, and (c) significant associated aortic or tricuspid valve disease.

Procedure

The procedure was performed via a transfemoral vein approach. Under sedation, right heart catheterization was performed. A pigtail catheter was kept in the aortic root as a marker. An interatrial septal puncture was made using the Brockenbrough needle. Needle entry into the left atrium was confirmed by injecting 2-3 ml of the contrast agent to confirm the left atrial entry. Once successful, a septal puncture was made, and unfractionated heparin was given intravenously, in a dose of 100 u/kg. After pushing the Brockenbrough sheath into the left atrium, the needle was withdrawn, and a coiled wire was then placed in the left atrium. A dilating sheath was passed over this wire. We used an Accura double-lumen balloon (Accura Medizintechnik GmbH, Karben, Germany). The balloon size was calculated by the formula height in centimetres / 10 + 10.

We started with an undersized balloon, 1-3 mm less than the calculated balloon size, to avoid injury to the mitral valve apparatus. The Accura balloon catheter was then advanced from the left atrium to the left ventricle across the mitral valve. After this, the distal part of the balloon was inflated with dilute contrast agent using a special graduated syringe. The balloon catheter was then pulled back, and its middle part was placed across the mitral valve. It was fully inflated with a preset amount of dilute contrast agent. When the waist in the middle of the balloon disappeared, it was rapidly deflated. Then, the pressure in the left atrium was measured, and if required, the procedure was repeated with incremental balloon sizes by filling it with a more dilute contrast solution. After the procedure, a final echocardiographic examination was done to confirm adequate mitral valve opening and to rule out any complications like the development of significant mitral regurgitation. The femoral access site was then managed using standard hemostasis techniques.

Statistical analysis

Data were analyzed using SPSS Statistics for Windows, version 26.0 (IBM Inc., Armonk, New York). Continuous variables are presented as mean ± standard deviation, and categorical variables as frequencies and percentages. Paired t-tests were used to compare pre- and post-procedural values, and the Chi-squared or Fisher's exact test was used for categorical comparisons. A p-value of <0.05 was considered statistically significant.

## Results

PBMV was performed on 46 patients. Twenty-three were males and 23 were females with rheumatic mitral stenosis, aged between nine and 17 years and seven months (Table 1).

**Table 1:** Baseline demographics

Variables	N=46 (%) / Mean ± SD
Male	23 (50%)
Female	23 (50%)
Age	14.35±3.24 years
Body surface area (m^2^)	0.97±0.16
Heart rate/min	81.2±43.4
Height (in cm)	138.4 ± 19.6
Weight (in kg)	24.9±9.1

A redo PBMV was done in one patient, in whom a prior PBMV was done. MS was severe in all cases, with MVA ranging from 0.57 cm^2^ to 0.93 cm^2^. All patients had a normal sinus rhythm, were in class II/III of the New York Heart Association (NYHA) symptom class, and none had evidence of left atrial thrombus or MV calcification. Seven patients had significant subvalvular pathology present. Mild mitral regurgitation was associated in 15(32.6%), aortic regurgitation was seen in six (13%), and tricuspid regurgitation in 13 (28.2%) children (Table 2). 

**Table 2:** Incidence of varying degrees of MR in pre-PBMV and post-PBMV status MR - mitral regurgitation, PBMV - percutaneous balloon mitral valvuloplasty

Mitral regurgitation	Pre PBMV N=46(%)	Post PBMV N=46(%)	1-month follow-up N=46(%)
No	31 (67.4%)	16 (34.8%)	5 (10.5%)
Mild	15 (32.6%)	23 (50%)	9 (19.6%)
Moderate	0	6 (13%)	6 (13%)
Severe	0	1 (2.2%)	1 (2.1%)

**Figure 1:**
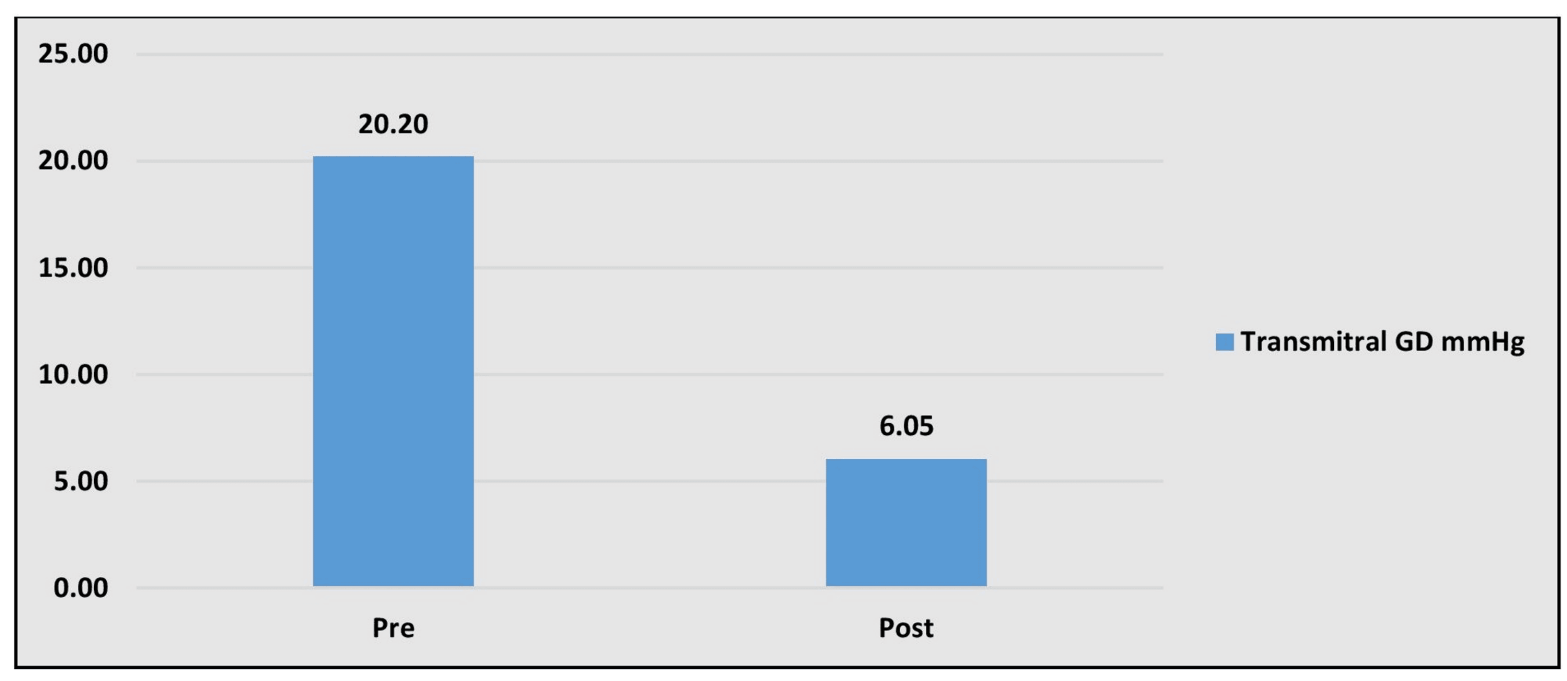
Graph showing mean transmitral gradient difference of pre and post PBMV procedure PBMV - percutaneous balloon mitral valvuloplasty

**Figure 2:**
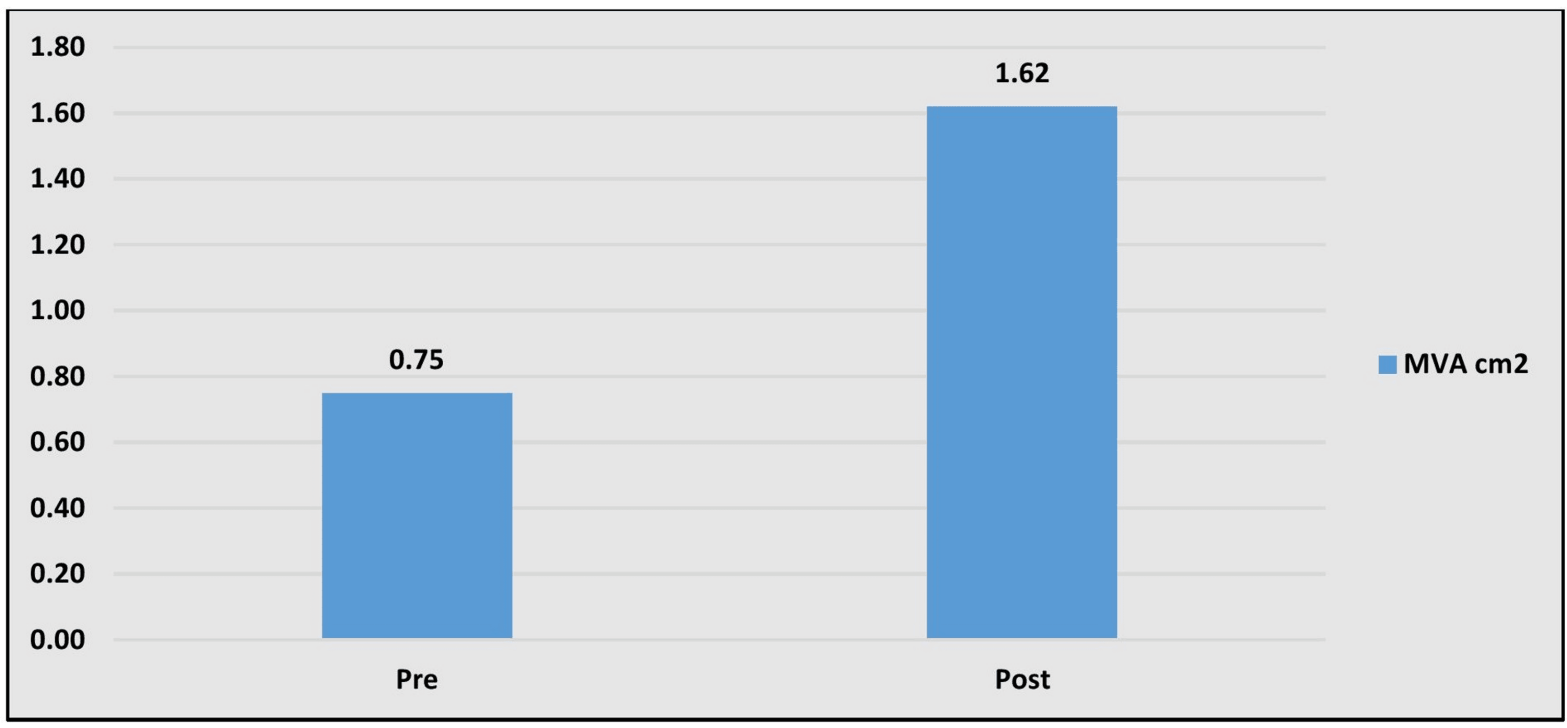
Graph showing mean MVA difference of pre and post PBMV procedure MVA - mitral valve area, PBMV - percutaneous balloon mitral valvuloplasty

Their heights ranged from 118 to 155 cm (mean 138.4±19.6 cm) and weights 16 to 36 kg (mean 24.9±9.1 kg) with a mean body surface area (BSA) of 0.97±0.16 m^2^ (range 0.70 to 1.25 m^2^). Our overall success rate was 97.8%. We used a smaller balloon in all the patients and had to upgrade in only seven cases based on significant residual gradients. There was a significant reduction in the transmitral gradient from 19.9±6.2 mmHg to 3.5±1.1 mmHg (p<0.001; Figure 1), and a significant increase in the mitral valve area from 0.75±0.1 cm^2^ to 1.6±0.2cm^2^ (p<0.001, Figure 2 and Table 3).

**Table 3:** Procedural outcomes LA - left atrial, PBMV - percutaneous balloon mitral valvuloplasty t-test statistics test was used for significance (<0.0001* showed statistical significance)

Variables	Pre-PBMV, mean±SD	Post-PBMV, mean±SD	p-value
Mitral valve area (cm^2^)	0.75±0.18	1.62±0.27	<0.0001*
Echocardiographic mean gradient (mmHg)	20.2±7.71	6.05±2.48	<0.0001*
LA pressure (mmHg)	24.12±10.02	15.77±8.93	<0.0001*
Catheterization gradient (mmHg)	19.93±6.21	3.54±1.1	<0.0001*
Pulmonary artery systolic pressure (mmHg)	48.71±20.93	33.11±10.75	<0.0001*

There was immediate symptomatic improvement in all patients, and there was no mortality. Severe MR developed in one patient. Out of seven patients, moderate MR developed in six patients, and only one had subvalvar pathology. Table 4 showed no statistically significant relationship between the undersizing of the balloon and the development of MR, although the incidence of moderate to severe MR was higher with increasing balloon diameters.

**Table 4:** Relationship between undersizing of balloon and development of MR MR - mitral regurgitation Chi-squared test was used for significance (<0.05 value showed statistical insignificance)

Degree of mitral regurgitation (MR)	0 mm N=4(%)	1mm N=11(%)	2mm N=13(%)	3mm N=12(%)	>3mm N=6(%)	p-value
No MR	0	1(9.1%)	1 (7.6%)	2 (16.7%)	1 (16.7%)	0.86
Trivial MR	0	0	2 (15.4%)	0	1 (16.7%)	0.33
Mild MR	2 (50%)	8 (72.7%)	8 (61.5%)	9 (75%)	4 (66.6%)	0.88
Moderate MR	1 (25%)	2 (18.2%)	2 (15.4%)	1 (8.3%)	0	0.74
Severe MR	1 (25%)	0	0	0	0	
Post procedure mean gradient (cm^2^)		7.5±2.5	6.10±1.5	5.91±3.02	5.33±2.07	0.25

This table evaluates the association between the extent of balloon undersizing (categorized as 0 mm, 1 mm, 2 mm, 3 mm, and >3 mm smaller than the height-based estimated size) and the severity of mitral regurgitation (MR) observed post-procedure. The table also provides the mean post-procedural gradient corresponding to each degree of undersizing, along with the p-values for intergroup comparisons, thereby demonstrating that no statistically significant relationship exists between the degree of undersizing and MR severity.

## Discussion

There has been a significant decrease in the incidence of rheumatic fever (RF) over the years, but despite that, it continues to affect young people, especially those from poor socioeconomic strata and those with little access to primary health care [1]. This highlights the ongoing need for effective and accessible treatment options for this population.

The results of this study show that PBMV is a safe and effective procedure for symptomatic mitral stenosis in the young population with a low rate of complications. Long-term results of PBMV show a sustained benefit in approximately 75% of cases [2]. This study demonstrates that PBMV results in a good immediate hemodynamic and clinical improvement in the majority of patients with mitral stenosis (~98%), which is similar to other studies.

Reduction of MVA and of the mean diastolic transmitral gradient with symptomatic relief confirmed the efficacy of this technique. There was a significant reduction in the transmitral gradient from 19.9 ± 6.2 mmHg to 3.5± 1.1 mmHg (p<0.001) and a significant increase in the area of the mitral valve from 0.75± 0.1 cm2 to 1.6 ± 0.2 cm2 (p<0.001).

It is noteworthy that PBMV involves low cost and has a rapid turnover time compared to surgery. Moreover, outcomes have been found to be comparable to surgery. In patients with rheumatic heart disease, the advantages of palliation and preservation of the native mitral valve over valve replacement surgery are well established [4,5]. Rheumatic disease is known to have a chronic and progressive course, and young patients are prone to undergo more than one valvular procedure during their lifetime. There are long-term implications of preserving the native valve, especially in a demographic likely to require future interventions. Therefore, whenever further interventions are required, surgery can be performed without the inherent risks of a previous thoracotomy.

However, before deciding on the preferred therapeutic modality, a complete morphologic assessment of the diseased valve should be performed with the subvalvular apparatus in focus. The subvalvular apparatus (SVA) is an important component of the mitral valve structural complex. Although PBMV is associated with subvalvular structures [6], significant subvalvular pathology has been known to be a risk factor for suboptimal results [7]. We had seven patients with subvalvular pathology (15%), all of whom achieved therapeutic success. Although Goswami et al. found that the extent of subvalvular deformity does not have a significant effect on the immediate outcome of mitral valvuloplasty and can be successfully performed in patients with severe subvalvular fibrosis, severe SVA deformity may impose technical difficulties and therefore affect immediate results [8].

There was immediate symptomatic improvement in all patients, and there was no mortality. Severe MR developed in one patient. Moderate MR developed in six patients. Among the seven patients who developed significant MR (six moderate and one severe), only one patient had concomitant significant subvalvular pathology. The main predictors of MR are oversizing of the balloon [9], commissural or leaflet calcification or both, and the intensity of the involvement of the mitral subvalvar apparatus [10]. Our index case did not have any of the risk factors mentioned.

Our PBMV results have been comparable to those of other recent studies. Shrestha et al in a recent study from Nepal on PBMV in children aged 7-15 years in 100 children had a 94% success rate, and they reported no complications [11]. Abdallah et al. in a study on 64 patients reported a success rate of 96.8%. Although the rates of severe MR were comparable, they also reported complications of cardiac tamponade and embolic stroke in 1.6% cases each [12]. In a large study from India, Kothari et al reported a 94% success rate in 100 children of the 7-12 year age group. They had an incidence of MR in two patients, and two had to be sent for surgery [13].

Our study also demonstrates that the results of PBMV in children and adolescents are comparable to those of adults. Karur et al. reported that PBMV yields superior outcomes in juveniles compared to adults, with larger post-procedural MVA and lower residual gradients [14]. Our findings are consistent with these observations. Similarly, Sinha et al. previously showed greater effectiveness of PBMV in the juvenile age group; hemodynamic benefits were found to be superior in young people, with mean PASP and PVR found to reduce more. In addition, this benefit was found to be sustained for a mean follow-up duration of 29 months [15]. More favourable results are likely due to more favourable anatomy in the younger population in the form of smaller LA size, pliable MV, and relatively less thickened atrial septum [16].

Although there are a lot of studies on PBMV in general, there is no consensus on the selection of balloon size. We used balloons 1-3 mm smaller than the size derived based on height criteria. We agree with Krishnamoorthy et al., who showed in their study that despite PBMV being an effective procedure in small children, a high incidence of moderate MR occurs, mainly because the choice of balloon size is made using an adult-style, height-based nomogram [17].

Therefore, separate nomograms for children are warranted. At the same time, differences in body habitus and skeletal deformities have been shown to impact balloon sizing and therefore Tastan et al, demonstrated in a group of 128 adult patients that the intercommisural diameter of the mitral valve in diastole can be measured as an alternative, and choosing balloon size based on that can decrease the risk of MR and is associated with better MVA [18]. When the maximum diameter of the diastolic annulus is used for balloon sizing, the balloon reference sizes have been found to be smaller than those obtained with the height-based formula [19], and therefore, performing PBMV with smaller balloon sizes based on the height formula is a prudent selection criterion to minimize complications [20]. 

Overall, this study adds to the growing body of literature on effectiveness of PBMV in young population, and underscores need for ongoing research in this area in the form of multi center studies, longer follow up studies and developing age appropriate balloon sizing nomograms, particularly in developing tailored treatment approaches based on patient age and morphological characteristics.

Limitations

This study is subject to several inherent limitations. First, its retrospective design may introduce selection bias and uncontrolled confounding factors. Second, as a single-center study, the findings may not be generalizable to other settings or populations. Finally, the absence of long-term follow-up limits our evaluation of the durability of the procedural benefits and the incidence of late complications. Future prospective multicenter studies with extended follow-up are necessary to validate these findings and to refine balloon sizing protocols in juvenile mitral stenosis.

## Conclusions

Our results indicate that PBMV is a safe and effective procedure in the juvenile population with rheumatic mitral stenosis when performed with balloons undersized by 1-3 mm relative to the height-based estimate. Our results show a significant reduction in transmitral gradient and a concomitant increase in mitral valve area, leading to symptomatic improvement in nearly all patients. Importantly, it yields comparable outcomes even in patients with subvalvular pathology. High success rate with minimal complications underscores the potential of PBMV. Our results also support the use of an undersized balloon strategy to mitigate the risk of significant MR while preserving the benefits of native valve, a consideration that is particularly important in a young patient population likely to require future reinterventions. 
